# Editorial for the Special Issue “Diagnosis and Treatment of Diseases of the Facial Skeleton, Oral Cavity, and Paranasal Sinuses”

**DOI:** 10.3390/medicina61122191

**Published:** 2025-12-11

**Authors:** Kamil Nelke, Maciej Dobrzyński, Marceli Łukaszewski

**Affiliations:** 1 Maxillo-Facial Surgery Ward, EMC Hospital, Pilczycka 144, 54-144 Wroclaw, Poland; 2 Academy of Applied Sciences, Health Department, Academy of Silesius in Wałbrzych, Zamkowa 4, 58-300 Wałbrzych, Poland; 3 Department of Pediatric Dentistry and Preclinical Dentistry, Wroclaw Medical University, Krakowska 26, 50-425 Wroclaw, Poland; 4 Department of Anaesthesiology and Intensive Care, Sokołowski Hospital, Sokołowskiego 4, 58-309 Wałbrzych, Poland

The scope of diseases and lesions that might be present and found in the facial skeleton, oral cavity, and paranasal sinus is very broad and wide-reaching. Each might have various etiological factors and a stage of local growth and development. When evaluating each patient’s local status, a good radiological evaluation, followed by patient anamnesis, accurate differential diagnostics, and adequate preparation for surgery, impact on the final results. Current trends in maxillo-facial surgery not only include the usage of patient individual solutions, virtual planning, and 3D-designed hardware, but also implementations in patient-selected surgical cases to improve patient outcomes and prognosis. Because some lesions tend to be more or less common in the facial skeleton area, the possible treatment methods and algorithms can differ greatly based on case, surgeon, and many other individual factors [1,2]. An intraoral approach is more common than an intranasal approach alone; however, a combination of intraoral approach with an endoscope is also used more commonly by maxillofacial surgeons [3,4,5]. CBCT planning makes approaching the sinus floor safer [3]. Possible local nerve paresis can be treated with good outcomes using pharmacological agents, especially vitamin B with or without alpha-lipoic acid oral intake, as suggested by some researchers [4].

At present, many jawbone and adjacent tissue lesions can be evaluated and diagnosed with CBCT (cone-beam computed tomography). The role of this imaging method is helpful in estimating the presence of some odontogenic or non-odontogenic lesions, as it carefully evaluates bone structure within tooth-bearing structures. The usage of AI (artificial intelligence) for pattern evaluation in CBCT is the next step. Studies like that of Al-Haj Husain and others confirm that CBCT focuses on not only jawbone structures and conditions, but also sinuses and their patency or spread of inflammation within [6]. Appropriate diagnosis helps identify the pattern of certain sinus lesions, as well as their boundaries and possible anatomical variations in future operable areas [6,7,8]. The study by Morawska-Kochman et al. indicates that airflow in the sinuses and nasal cavity might experience disturbances stemming from both anatomical and post-operative factors [9].

Studies, like those by Mclean et al. and Omami and Yeoh, summarize the occurrence and possible patterns and characteristics of various typical jaw cysts and tumors that can be found during radiological and clinical patient evaluation [10,11]. It is worth noting that the radiolucent, radiopaque, or mixed appearance of each bone lesion could be characteristic of some tumor of odontogenic, non-odontogenic, or other bone lesion abnormalities. The authors in this Special Issue emphasize the role of individual case-related studies and planning the scope of each surgery based on 3D models. Sometimes, CBCT can be useful, detecting incidental tumors or lesions that are not found under normal circumstances. The usage of CT and CBCT is important for assessing disturbances in nasal airflow and smell sensation and distinguishing between an odontogenic-like lesion and fibrous dysplasia, as presented in Resler et al.’s case report [12].

On the other hand, the scope of maxillary sinus radiological imaging and all paranasal sinus diseases and their conditions might have different possible etiological factors, including odontogenic and non-odontogenic sources, where odontogenic sinusitis (ODS) plays a role in the majority of related cases [13,14]. A recent review by Craig (2022) indicates that collaboration between general dentists, laryngologists, and maxillofacial surgeons could benefit the treatment and patient outcomes when all aspects of ODS are treated equally using a team-based approach [14]. Perhaps future improvements might include a combination of simultaneous FESS-CBCT (functional endoscopic sinus surgery–cone beam computed tomography) navigation for not only ODS but also minimally invasive sinus surgery. All diagnostic approaches should also take into consideration that the area of the maxillary alveolar process and maxillary sinuses can be not only occupied by bone anomalies like fibrous dysplasia, ossifying fibromas, cemento-ossifying lesions, but also more aggressive lesions like squamous cell carcinoma or rhabdomyosarcoma, as demonstrated in the studies by Resler et al. and Mârțu et al. [12,15]. As elucidated in the following sections, each lesion requires sufficient diagnoses and tumor sampling to establish the most accurate approach for each patient.

Currently, all lesions and diseases of the jaw bones, sinuses, and surrounding tissues can be treated using many different approaches. Some important aspects include studying the expression of certain markers to determine patients’ outcomes and the necessity and scope of each surgery. Mostafa et al. investigate the expression of glucose transporter-1 in oral epithelial dysplasia, while Mahamad Apandi et al. examine the expression of mucin; the results of these studies may help both histopathologists and surgeons understand how certain lesions are diagnosed, progress in time, and what treatment is more suitable in those cases [16,17].

The three-dimensional proportions and relations between the lower, middle, and upper areas of the face and skull also influence decision-making. In the most common dento-alveolar skeletal malocclusion cases, which can be treated with great overall success with different orthognathic surgery steps, it is also worth remembering that the scope of CBCT and 3D planning can affect any other different surgeries that include feminization and masculinization procedures, with goals that are mostly related to facial beauty and esthetics [18,19]. Both surgical scopes depend on individual 3D planning protocols, surgical guides, 3D models, and individual PEEK or other implants; however, Beyer et al. conclude that precise surgical guides promote better, more accurate, and predictable results with less unstable long-term results [19]. Individually designed and manufactured 3D-printed models may take less time when new dental materials include 3D-bone-like meshes, which are now quite commonly used to evaluate and plan cis- and trans-patients’ surgeries, establishing the scope of surgery by improving patients’ outcomes, satisfaction levels, and quality of life. Recent studies by Pokrowiecki et al. highlighted a possible new methodology for the following surgeries [20,21].

Each pathology can be treated with many possible methods and approaches; however, some interesting cases can open new possibilities and treatment modalities or present a novel or atypical scope of the disease. Paranasal sinus pathologies are not only related to cysts, tumors, or lesions; the scope of possible teeth-related and odontogenic origins should also be considered. Some more aggressive lesions might include oral cancer spreading towards the sinuses, the occurrence of small and major salivary gland tumors, or the presence of sarcomas, vascular tumors, neurogenic tumors, and other conditions that can be found in the facial skeleton area [22,23]. Some approaches, including neuronavigation and computed tomography, should be used simultaneously in the virtual reality environment to minimize the risk of any real in vivo surgery. In each surgery, care should be taken to reduce damage, luxation, trauma, or temporo-mandibular joint overload. A recent review by Yoshida highlights the important aspect of TMJ dislocation [24]. On the other hand, any joint or mandibular asymmetries, problems with occlusion, TMJ acoustic symptoms, and possible related malocclusion should always be differentiated from unilateral condylar hyperplasia (UCH) [25].

In conclusion, a majority of surgical procedures in the facial skeleton area can influence patients’ quality of life; improve bite, chewing, and function; and enhance esthetics and overall patient confidence. Improvements in healthcare and new technologies, specifically diagnostic approaches used in different jawbone and facial skeleton surgeries, are promising for the future of more specialized maxillofacial surgery.

We believe that these eleven contributions improve our current understanding of certain fields, i.e., oral, jawbone, maxillary sinus, and related facial skeleton diseases, and have the potential to stimulate further research.
